# Renal metastases from esophageal cancer and retroperitoneal lymphoma detected via chromosome duplications identified by fluorescence in situ hybridization in urine exfoliated cells: First 2 case reports

**DOI:** 10.1097/MD.0000000000024010

**Published:** 2021-03-12

**Authors:** Zhiquan Hu, Chunjin Ke, Yuanqing Shen, Xing Zeng, Chunguang Yang

**Affiliations:** Department of Urology, Tongji Hospital Affiliated Tongji Medical College of Huazhong University of Science and Technology (HUST), 1095 Jiefang Avenue, Wuhan, China.

**Keywords:** renal metastasis, esophageal cancer, lymphoma, fluorescence in situ hybridization, case report

## Abstract

**Rationale:**

Renal-occupying lesions positive for urine fluorescence in situ hybridization (FISH) are usually considered urothelial carcinomas. Here, we describe 2 cases of renal metastases with chromosome duplications in urine exfoliated cells.

**Patient symptoms:**

Patient 1, a 56-year-old male with a history of esophageal cancer, was admitted to our hospital on May 2017 after presenting with right back pain with microscopic hematuria for 1 month. Magnetic resonance imaging (MRI) showed right renal space-occupying lesions (5.4 cm × 4.6 cm) and multiple enlarged lymph nodes in the right renal hilum and retroperitoneum. The cystoscopy results were negative, and FISH analysis of urine exfoliated cells was positive, indicative of chromosome 3, 7, and 17 amplifications. Patient 2 was a 50-year-old male who was admitted to our hospital on May 2019 with no obvious cause of abdominal pain and abdominal distension (lasting for 7 days), with a serum creatinine level of 844 μmol/L. Patient 2 had no hematuria or fever, and MRI showed left renal inferior and medial space-occupying lesions, and multiple mesenteric nodules at the junction of the left adrenal gland, retroperitoneum, abdomen, and pelvis, which were partially fused. The tumor lesions were approximately 3.1 cm × 2.3 cm in size. The urine FISH results were positive, indicating chromosome 3, 7, and 17 amplifications.

**Diagnoses:**

Both patients were diagnosed with renal tumors with unknown pathology.

**Interventions:**

Patient 1 underwent laparoscopic resection of the kidney and ureter, and sleeve cystectomy. The postoperative pathological diagnosis was metastatic keratinized squamous cell carcinoma, with squamous cell carcinoma in the right hilar lymph node. Histological FISH of the primary esophageal cancer and renal metastases were consistent with the urine FISH test results. Patient 2 underwent a biopsy of the left renal inferior and retroperitoneal areas, and was diagnosed with diffuse large B-cell lymphoma.

**Outcomes:**

Patient 1 survived 6 months after urological surgery. After treating patient 2 with the R-CHOP regimen and kinase inhibitors, his renal function recovered significantly and the mass become undetectable.

**Lessons:**

Our results imply that FISH-positive renal occupying lesions should be considered as potential renal metastases with chromosome aberrations when making a differential diagnosis.

## 1 Introduction

The incidence of renal metastases is low, and Klinger et al^[1]^ reported 118 cases of renal metastases (including lymphoma) in 5000 autopsies (2.4%). When lymphoma was excluded, 73 patients had renal metastases at autopsy, accounting for 1.5% of the total population. Data from a series of autopsy studies^[1–4]^ showed that the most common primary tumors that metastasized to the kidney were lung cancer (19.8%–23.3%), breast cancer (12.3%), and gastric cancer (11.1%–15.1%). Renal blood flow is abundant, accounting for close to 20% to 25% of the cardiac output. The primary tumor is often transported to the kidneys by blood metastasis. Genetic abnormalities are found in almost all tumors and have been used for diagnosing and determining the malignancy of many types of tumors. Urovysion fluorescence in situ hybridization (FISH) is a sensitive and specific method for diagnosing urothelial carcinoma. It has been approved for screening patients with hematuria and monitoring for recurrent urothelial carcinoma. Compared with conventional cytology, FISH has a higher sensitivity for detecting urothelial carcinoma (81% vs 58%).^[5]^

This case report focuses on data for 2 patients with renal metastases who were admitted to our hospital with a positive urine FISH test. According to previous literature, this is the first report of renal metastasis detected by urine FISH testing. We reviewed the relevant literature to explore the reason why FISH technology could be used successfully to identify renal metastases and reduce the rate of misdiagnosis of renal metastases.

## 2 Case reports

**Figure 1:**
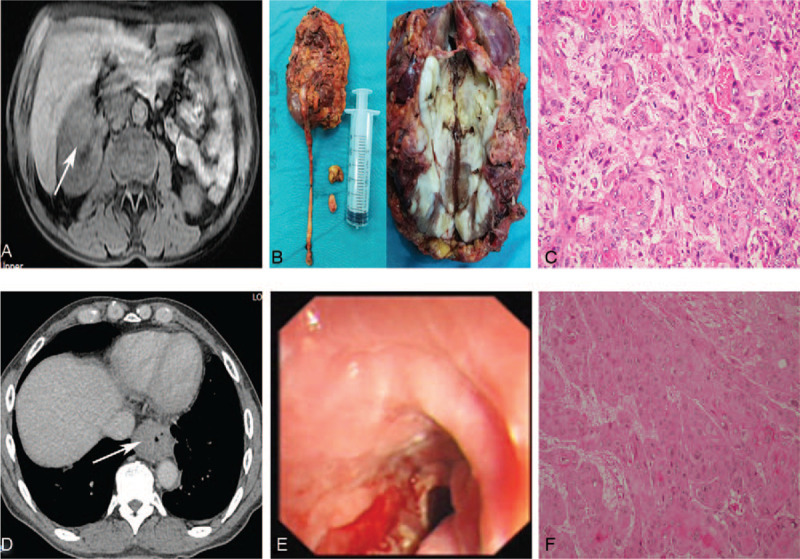
Clinical manifestations of patient 1. (A) Magnetic resonance imaging (MRI) scan of the renal metastases. The tumor location is indicated by the arrow. (B) Postoperative specimens of the renal metastases. (C) Postoperative pathology of the renal metastases. Microscopy revealed metastatic keratinizing squamous cell carcinoma (hematoxylin and eosin staining; magnification, ×200). (D) Computed tomography scan of the primary esophageal cancer. The tumor location is indicated by the arrow. (E) Gastroscopic view of the primary esophageal cancer. (F) Postoperative pathology of the primary esophageal cancer. Microscopy revealed highly-to-moderately differentiated esophageal squamous cell carcinoma (hematoxylin and eosin staining; magnification, ×200).

**Figure 2:**
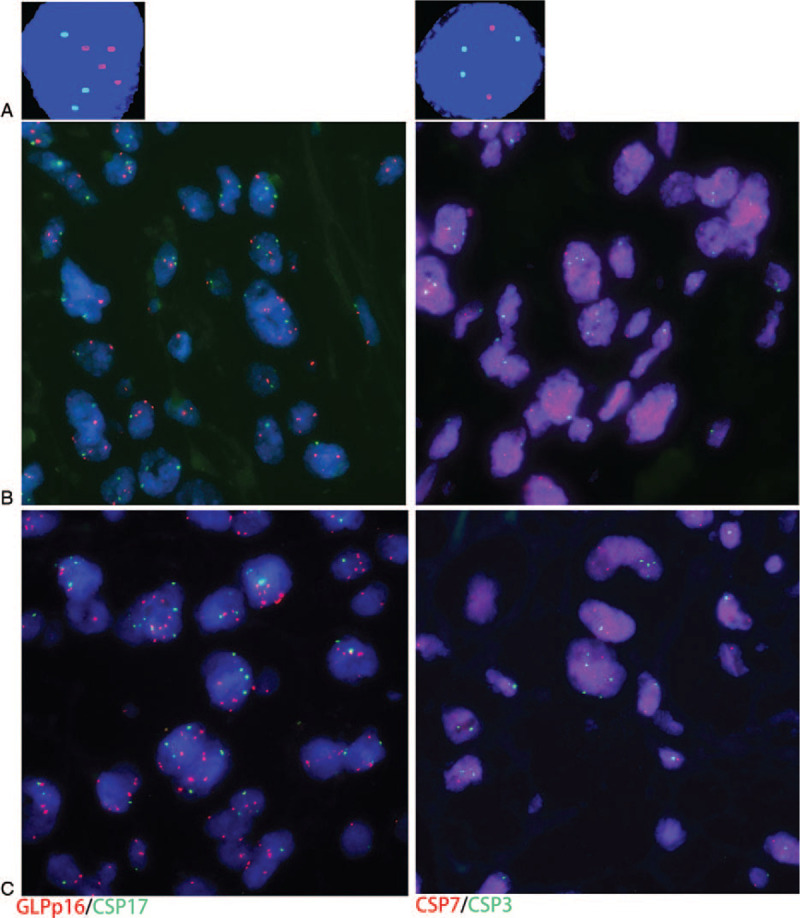
Cytological and histological fluorescence in site hybridization (FISH) analysis for patient 1. (A) Cytological FISH analysis of cells shed into the urine. (B) Histological FISH analysis of the primary esophageal cancer tissue. (C) Histological FISH of the renal metastases. Red fluorescence represents CSP7 and GLPp16, and green fluorescence represents CSP7 and CSP17.

Patient 1, a 56-year-old male, was admitted to our hospital on May 2017 after presenting with right back pain with microscopic hematuria for 1 month. Upon admission, he had diarrhea with low-grade fever and no frequent urination or urgency of urination. No other abnormalities were observed during the physical examination. Magnetic resonance imaging (MRI) showed right renal space-occupying lesions (5.4 cm × 4.6 cm) and multiple enlarged lymph nodes in the right renal hilum and retroperitoneum, the larger of which was 2.3 cm × 1.8 cm. These lesions may have been renal pelvis cancer invading the renal parenchyma (Fig. 1A). The cystoscopic results were negative, and the FISH results of urine exfoliated cells were positive, indicating the presence of chromosome 3, 7, and 17 amplifications (Fig. 2A). The patient's previous medical history was as follows. In 2016, the patient was admitted to the Department of Thoracic Surgery of our hospital for progressive eating obstruction, which had persisted for 2 years. A computed tomography (CT) scan suggested obvious thickening of the lower esophageal wall, stenosis of the lumen, and the possibility of neoplastic lesions (Fig. 1D). Gastroscopy and ultrasound endoscopy revealed esophageal cancer (T2-3N1-x; Fig. 1E), and the patient then underwent radical surgery for resection of esophageal cancer under general anesthesia in March 2016. The postoperative pathological diagnosis was highly-to-moderately differentiated squamous cell esophageal carcinoma invading the whole wall of the esophagus, with some positive lymph nodes (Fig. 1F).

Patient 1 underwent laparoscopic resection of kidney and ureter, and sleeve cystectomy (Fig. 1B). The postoperative pathological diagnosis was metastatic keratinized squamous cell carcinoma, and squamous cell carcinoma was seen in the right hilar lymph node. These findings, combined with the patient's medical history, led to a diagnosis of esophageal squamous cell carcinoma metastasis (Fig. 1C). In addition, we also performed histological FISH analysis of the patient's primary esophageal cancer tissue and renal metastatic tumor tissues. The procedures used for the histological FISH testing were previously described by Michelle et al. in a report on the application of FISH to nonurothelial carcinoma^[6]^. The FISH results were analyzed in a double-blinded manner by 2 professional certified pathologists, who had worked in the field for 10 years. At least 25 morphologically abnormal cells were analyzed. Four cells contained multiple chromosomes (more than 1 probe for chromosomes 3, 7, and 17 had 3 or more signals), and 12 cells were positive for a gene locus-specific probe p16 [GLPp16] deletion. The histological FISH results of the primary esophageal cancer tissue (Fig. 2B) and the renal metastatic tumor tissue (Fig. 2C) were positive, and these findings suggested the presence of chromosome 3, 7, and 17 amplifications, but no other abnormalities, which was consistent with the urine FISH test results (Fig. 2). Patient 1 survived 6 months after urological surgery.

**Figure 3:**
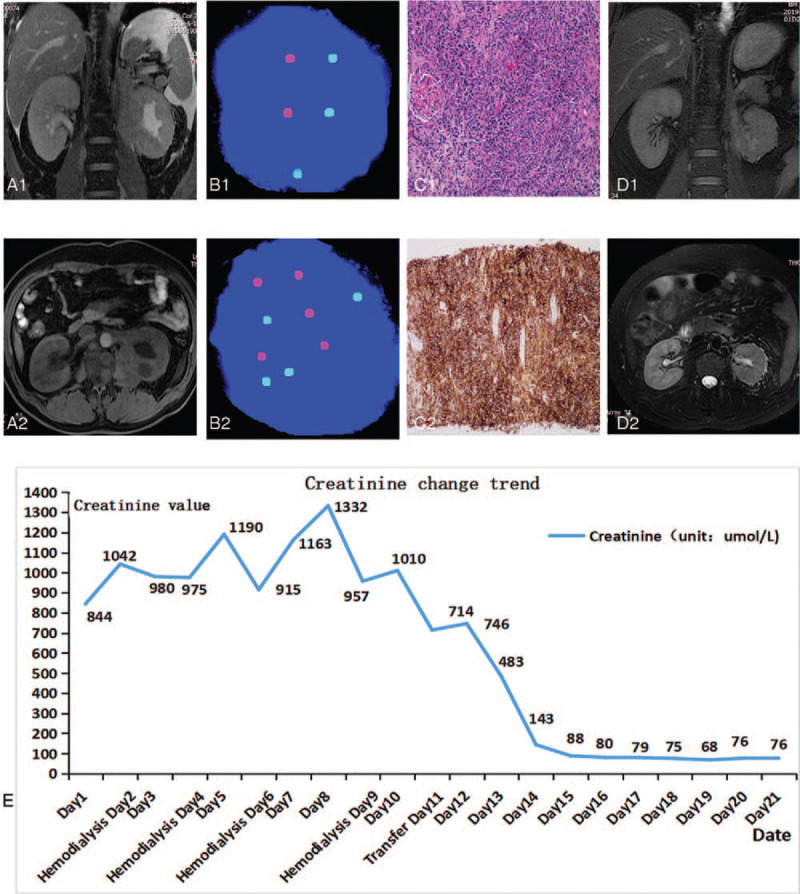
Clinical manifestations of patient 2. (A1 and A2) Magnetic resonance imaging scan at admission. (B1 and B2) fluorescence in site hybridization-positive exfoliated cells in the urine. Panel B1 shows chromosome 17 (green) amplification, and panel B2 shows chromosome 3 (green) and 7 (red) amplification. (C1) Hematoxylin and eosin staining of the biopsy sample (×200) was consistent with non-Hodgkin B-cell lymphoma (invasive), likely diffuse large B-cell lymphoma. (C2) CD20 staining was positive by immunohistochemistry. (D1 and D2) Magnetic resonance imaging after 8 cycles of chemotherapy. (E) Changes in creatinine levels between the first day of admission and transfer to our hematology department.

Patient 2 was a 50-year-old male who was admitted to our hospital on May 2019 with no obvious cause of abdominal pain and abdominal distension, which had lasted for 7 days, without an explanatory medical history. The patient had no hematuria or fever, and the MRI showed left renal inferior and medial space-occupying lesions, and multiple mesenteric nodules at the junction of the left adrenals, retroperitoneum, abdomen, and pelvis, which were partially fused. The tumor lesions were approximately 3.1 cm × 2.3 cm in size, and further puncture biopsy was recommended (Fig. 3A1 and 3A2). The urine FISH results were positive, indicating chromosome 3, 7, and 17 amplifications (Fig. 3B1 and 3B2). The laboratory examination revealed a creatinine level of 844 μmol/L, an estimated glomerular filtration rate (eGFR) of 5.7 ml/(minute 1.73 m^2^), and a blood potassium level of 5.12 mmol/L (the reference range at our hospital is 3.5 mmol/L–5.1 mmol/L). The patient underwent hemodialysis every 2 days in the dialysis room, but his renal function remained very poor. After routine preparation, biopsies of the left renal inferior and retroperitoneal areas were performed under local anesthesia with the guidance of B-ultrasound. The postoperative pathological diagnosis was non-Hodgkin B-cell lymphoma (invasive), consistent with diffuse large B-cell lymphoma (DLBCL; Fig. 3C1). The immunohistochemistry results were as follows: CD20 (L26) (+), CD20 (positive control) (+) (Fig. 3C2), CD19 (+), CD22 (+), PAX-5 (SP34) (+), CD19 (+), CD5 (+), CD43 (slightly +), CD21 (+), LEF (+), HGAL (+), BCL-2 (SP66) (+), BCL-6 (+), P53 (+), and C-myc (approximately 40% +). The tumor had invaded the kidney tissue. The patient was then transferred to the hematology department for further treatment. The hematologic diagnosis was phase III DLBCL, non- germinal-center B cell-like, and the Eastern Cooperative Oncology Group score was 2. The treatment provided in the hematology department was based on the R-CHOP treatment regimen combined with kinase inhibitors. After 8 cycles of chemotherapy, the patient's renal function recovered significantly, which was a better result than that achieved with hemodialysis (Fig. 3E). The mass shrank and became undetectable by radiography (Fig. 3D1 and 3D2).

This study was reviewed and approved by the Medical Ethical Committee of Tongji Hospital of Huazhong University of Science and Technology.

## 3 Discussion

The renal gland is the fifth most common site of malignant tumor metastasis after the lungs, liver, bone, and adrenal glands.^[7]^ Renal metastases have no obvious clinical symptoms: <20% of patients have microscopic hematuria, and only 5% develop renal failure. Therefore, it is often difficult to reach and confirm the diagnosis in time, and its occurrence indicates that the primary tumor has disseminated, and the prognosis is poor.

Patient 1 had a solitary metastasis in the right kidney that originated from esophageal cancer. Esophageal cancer is mainly divided into squamous cell carcinoma and adenocarcinoma, of which squamous cell carcinoma accounts for 80% to 90% of all cases. Previous findings showed that squamous cell carcinoma is more likely to metastasize to the kidney than to other locations.^[8]^ Metastases of esophageal cancer to the kidney are considered to be very rare, especially unilateral isolated renal metastases.^[9]^ Upon imaging, it is difficult to distinguish whether the tumor represents primary renal pelvis transitional cell carcinoma or metastatic renal pelvis carcinoma because both diseases show infiltrative growth into the renal parenchyma and multiple protrusions into the renal tissue, and some cases are accompanied by invasion of the perirenal fat or perirenal tissue.^[10]^ In such cases, the renal metastases are usually treated as a primary renal tumor, and nephrectomy is recommended. At present, the literature contains few related case reports. Due to the rarity of cases, the European Association of Urology guidelines and the National Comprehensive Cancer Network guidelines have not provided exact treatment methods.^[11]^ Grise et al^[12]^ proposed that for patients with solitary renal metastasis, radical nephrectomy can be performed under good physical conditions. However, the median survival time of patients with esophageal cancer that had metastasized to the kidney is only 2 to 10 months,^[13]^ and the survival time of our patient after urological surgery was 6 months. Therefore, we need improved methods for early detection and more effective systemic treatments to improve the disease-free survival of such patients.

Patient 2 had renal lymphoma, which can be divided into primary and secondary renal lymphoma. This patient had a secondary renal lymphoma according to our MRI findings and immunohistochemistry. The kidney itself does not contain lymphoid tissue, and the chance of lymphoma originating in the kidney is very small, accounting for only 0.7% of extranodal lymphomas.^[14]^ Renal lymphoma mainly develops secondarily to other diseases, mainly non-Hodgkin lymphoma (NHL). Autopsies of patients with lymphoma have suggested that the probability of the kidney being involved is as high as 50%,^[15]^ but the clinical manifestations lack specificity. The presurgery detection rate in patients was reported to be as low as 0.8%.^[16]^ Renal NHL can cause clinical manifestations resembling those of renal parenchymal diseases, such as acute renal failure, hematuria, and proteinuria due to tumor compression, interstitial infiltration, and intraglomerular infiltration. Renal changes are mainly caused by blood dissemination and the direct invasion of retroperitoneal lesions.^[17]^ Kidney invasion is a late manifestation. By that time, many other organs are involved, which can manifest as large, fused lymph nodes of uniform density. The MRI scan of patient 1 showed multiple mesenteric nodules at the junction of the left adrenal gland, retroperitoneum, and abdominal pelvic cavity, which were partially fused. The clinical manifestations of renal secondary lymphoma are not specific, which may lead to missed diagnoses and misdiagnoses. Such misdiagnoses affect the stage assigned to the disease at diagnosis and, thus, the choice of treatment plan and the prognosis. Some patients may have renal dysfunction or even renal failure. Patient 1 was found by physical examination to have no obvious abdominal pain or abdominal distention. At that time, the creatinine value was 844 μmol/L, and the eGFR was 5.7 ml/(minute 1.73 m^2^), which are values that correspond to renal failure. Therefore, renal involvement is usually silent clinically. The diagnosis of renal NHL depends on the pathological examination of the biopsy specimen. The histological type, DLBCL, is more common than renal NHL. Patient 1 had renal NHL, which is mainly treated by chemotherapy, and impaired renal function is an important factor in the poor prognosis of NHL.

Subsequent analysis was performed to determine why the urine FISH results of both patients were positive. Chromosomal aberrations are a prominent feature of human malignancies. Most solid tumors exhibit complex cytogenetic abnormalities. The FISH DNA probes used at our hospital are a combination of a centromere probe and a site-specific recognition probe (Beijing Jinpujia Medical Technology Co., Ltd.) consisting of 2 combinations of Chromosome Region-Specific Probe 3 [CSP3 (green)/CSP7 (red) and GLPp16 (red)/CSP17 (green). Wu et al^[18]^ and Haisley et al^[19]^ observed decreased DNA copy numbers of chromosomes 4P, 5q, 6q, 9, 10p, 12p, 13, 14P, 15p, 18p, 18q, 20, 22, and Y in patients with esophageal squamous cell carcinoma. Chromosome gain and translocation occurred in all or part of chromosomes 1, 2p, 3, 4P, 5p, 5q, 6p, 7, 8, 10q, 11, 12q, 14q, 16, 17q, 19, and XP. Seven derived chromosomes (5, 8, 12, 14, 14, 14, and 17) showed complex translocations, each involving 3 or 4 chromosomes. Data from a series of studies^[20–22]^ showed that in NHL, the chromosome number abnormalities were mainly sub-diploid, pseudodiploid, super diploid, and sub-tetraploid, with a wide range of chromosome numbers ranging from 39 to 97, and the abnormalities mostly involved chromosomes 1, 3, 4, 5, 7, 8, 11, 14, 17, and 21. The main chromosomal structural aberrations that occurred were 1q+, 1p+, 6q−, 8q+, 14q+, 18p+, 18q+, and 2p21-p23. It was also found that changes in chromosome numbers and structures were closely related to patient disease-free survival and overall survival. FISH has also been used for differentially diagnosing lymphoma and detecting gene deletions, amplifications, and rearrangements, as these changes are closely related to the prognosis of patients. For example, high expression of the B-cell lymphoma (BCL)-6 and BCL-2 genes indicates that the prognosis of DLBCL is poor.^[23]^ The immunohistochemistry results of patient 2 indicated positivity for BCL-2 and BCL-6, which implied that the prognosis was poor.

The data from these studies indicate that tumor cells of esophageal squamous cell carcinoma and NHL have possible chromosome 3, 7, and 17 aberrations and/or deletion or amplification of the p16 gene locus on chromosome 9. If tumor cells transfer to the kidney and invade the renal parenchyma and collection system, and can transfer to the urine in sufficient quantities, then FISH analysis of the urine may be positive. However, FISH is a sensitive and specific method for diagnosing urothelial carcinoma. For the diagnosis of renal metastases, urine FISH positivity can be misleading. For example, examining the MRI of patient 1 suggested a right kidney-occupying lesion (5.4 cm × 4.6 cm). The neoplastic lesion may have resulted from renal pelvis cancer invading the renal parenchyma, leading the urine FISH to be positive. In the absence of the patient's medical history, we could have easily misdiagnosed the lesion as primary urinary urothelial carcinoma before surgery and assigned the patient an inappropriate treatment plan. The examination of the MRI of patient 2 showed left subserosal and left renal medial lesions, and multiple mesenteric nodules at the junction of the left adrenal gland, retroperitoneum, and abdominal pelvic cavity, which were partially fused. The tumor lesions were approximately 3.1 cm × 2.3 cm in size. The creatinine value was 844 μmol/L, and the eGFR was 5.7 ml/(minute 1.73 m^2^). Although the FISH result was positive, a renal biopsy guided by B-ultrasound and bone biopsy confirmed the presence of lymphoma, thereby avoiding an unnecessary surgery. FISH positivity does not necessarily reflect cancer of the urinary system and sometimes interferes with diagnosis, especially in cases of secondary tumors.

To conduct FISH analysis of nonurothelial cancers, many researchers have used a single urine cytology specimen or postoperative histological specimens. However, these methods cannot confirm the consistency of results obtained when using histological FISH and cytological FISH. It is impossible to rule out incorrect conclusions due to operational errors, interpretation errors, false positives due to inflammatory reactions, or other pathological changes. In this study, urine cytology and histology samples were used to simultaneously confirm the results in both sample types. The FISH test results of the primary esophageal cancer tissue and the renal metastatic tumor tissue in patient 1 showed aberrations in chromosome 3, 7, and 17, which were consistent with the urine FISH results. We confirmed that the tumor cells shed into the urine originated from the metastatic renal tumor tissue and that the cells were derived from esophageal cancer metastasis, rather than primary squamous cell carcinoma of the kidney, which complemented previous research. Patient 2 underwent a puncture biopsy, although the sample tissue was insufficient to perform histological FISH detection. However, after the patient received 8 cycles of lymphoma treatment in the hematology department, a comparison of the MRI scans and renal function before and after treatment showed that the patient had a very marked improvement, which further confirmed that the patient's positive FISH result was caused by lymphoma.

In summary, diagnosing renal metastases is difficult. It is necessary to increase the awareness of urologists that FISH may have positive manifestations in renal metastases and nonurothelial carcinomas, so as to reduce the misdiagnosis rate.

## Author contributions

**Conceptualization:** Chunguang Yang.

**Data curation:** Chunjin Ke, Yuanqing Shen.

**Formal analysis:** Zhiquan Hu, Chunguang Yang.

**Funding acquisition:** Chunguang Yang.

**Investigation:** Yuanqing Shen.

**Methodology:** Zhiquan Hu, Chunguang Yang.

**Project administration:** Zhiquan Hu.

**Resources:** Chunguang Yang.

**Software:** Xing Zeng.

**Supervision:** Xing Zeng.

**Validation:** Chunguang Yang.

**Visualization:** Chunjin Ke.

**Writing – original draft:** Chunjin Ke.

**Writing – review & editing:** Zhiquan Hu, Chunguang Yang.
